# Removal of lollipop-shaped stent-stone complex using direct-vision laser lithotripsy

**DOI:** 10.1055/a-2644-7982

**Published:** 2025-07-29

**Authors:** Shuping Wang, Xinying Tang, Bing Hu, Kunke Wang, Lijun Xu, Daojian Gao

**Affiliations:** 1 535219 Department of Endoscopy, The Third Affiliated Hospital of the Naval Medical University, Shanghai, China; 2 535219 Department of Gastroenterology, The Third Affiliated Hospital of the Naval Medical University, Shanghai, China

Long-term retention of biliary plastic stents (PS) can lead to a rare complication: the formation of a lollipop-shaped stent-stone complex (LSSC) 1 , where stones encase the proximal end of the stent, making endoscopic removal technically difficult. We report a case of successful LSSC removal using peroral cholangioscopy (POCS)-guided frequency-doubled dual pulse Nd:YAG (FREDDY) laser lithotripsy.

A 72-year-old man, previously diagnosed with IgG4-related sclerosing cholangitis, underwent biliary and pancreatic PS placement three years prior to alleviate obstructive jaundice ( Fig. 1 ), followed by corticosteroid therapy. He was lost to follow-up. One month before admission, he presented with jaundice and fever. Emergency ERCP showed resolution of the biliary stricture but revealed a retained LSSC in the common bile duct (CBD). Removal attempts using standard tools failed, fracturing the stent at the duodenal lumen. A supplementary PS was placed to ensure drainage ( Fig. 2 ). Following stabilization, repeat ERCP was performed using POCS-guided laser lithotripsy. The PS was fully encased in yellow stones, forming an LSSC. FREDDY laser lithotripsy (U-100 Plus; World of Medicine, Berlin, Germany) was applied at the stent-stone interface under direct visualization to fragment the stones and gradually release the stent. The stent was successfully removed, and residual stones were cleared ( Fig. 3 , Video 1 ). The patient recovered well and was discharged three days later.

**Fig. 1:**
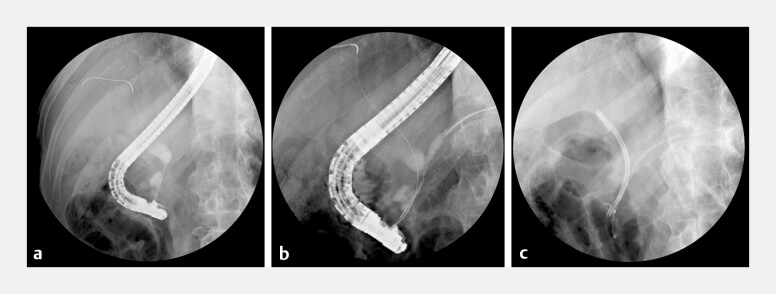
Initial ERCP findings (3 y prior). **a** Cholangiography showed a smooth, symmetrical stricture in the pancreatic segment of the common bile duct with upstream dilation. **b** Pancreatography revealed a slender, smooth stricture in the pancreatic head region of the main pancreatic duct, with mild dilation in the body and tail. **c** Plastic stents were placed in both the bile and pancreatic ducts.

**Fig. 2:**
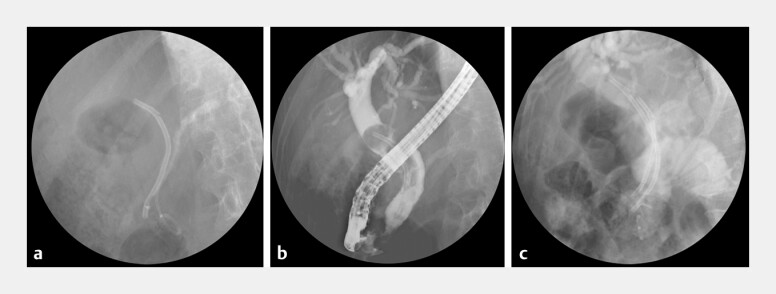
Emergency ERCP findings (1 mo prior). **a** Biliary and pancreatic plastic stents were seen in situ, with cast-like pancreatic duct stones along the pancreatic stent. **b** Cholangiography revealed a round filling defect encasing the stent, forming a lollipop-shaped stent-stone complex. **c** A second biliary stent was placed alongside the original.

**Fig. 3:**
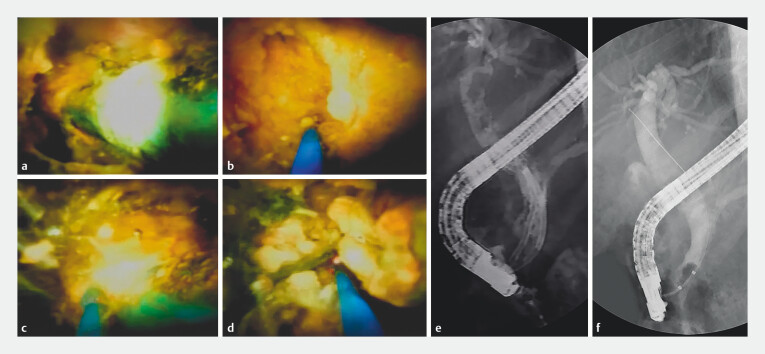
POCS-guided laser lithotripsy for LSSC removal. **a** POCS visualized a blue plastic stent encased in yellow stones, forming an LSSC. **b, c** A laser fiber was applied to the stent-stone interface and large stone. **d** Lithotripsy successfully fragmented the stone. **e** Cholangiography confirmed LSSC resolution and stone fragmentation. **f** Both stent and stone fragments were removed; balloon-occluded cholangiography showed no residual filling defects.

LSSC formation is associated with long-term stent retention (≥301 days) and CBD dilation 2 . The potential for LSSC formation should be considered in patients with long-term PS indwelling and dilated CBD. Endoscopic LSSC removal is technically challenging: standard endoscopic tools may fail, and forcible extraction risks ductal injury and perforation. Extracorporeal shock wave lithotripsy is technically complex 3 , while surgical intervention is traumatic. FREDDY laser lithotripsy is safer than Ho:YAG laser and electrohydraulic lithotripsy 4 5 . POCS-guided FREDDY laser lithotripsy offers a safe, minimally invasive, and effective approach for LSSC management.

Removal of lollipop-shaped stent-stone complex using direct-vision laser lithotripsy.Video 1

Endoscopy_UCTN_Code_CPL_1AK_2AF

## Notes

Endoscopy E-Videos https://eref.thieme.de/e-videos
*E-Videos* is an open access online section of the journal *Endoscopy* , reporting on interesting cases and new techniques in gastroenterological endoscopy. All papers include a high-quality video and are published with a Creative Commons CC-BY license. Endoscopy E-Videos qualify for HINARI discounts and waivers and eligibility is automatically checked during the submission process. We grant 100% waivers to articles whose corresponding authors are based in Group A countries and 50% waivers to those who are based in Group B countries as classified by Research4Life (see: https://www.research4life.org/access/eligibility/ ). This section has its own submission website at https://mc.manuscriptcentral.com/e-videos .
